# Pontocerebellar Hypoplasia Type 9: A Case Study Highlighting Distinctive Magnetic Resonance Imaging Features

**DOI:** 10.7759/cureus.58522

**Published:** 2024-04-18

**Authors:** Valentina Munera, Verónica Jaramillo, Brayan Muñoz-Caicedo, Sergio Vargas

**Affiliations:** 1 Department of Radiology, Universidad de Antioquia, Medellín, COL; 2 Department of Pediatrics, Universidad de Antioquia, Medellín, COL; 3 Department of Radiology, CediMed, Medellín, COL

**Keywords:** neuroimaging, magnetic resonance imaging, periventricular leukomalacia, pediatric posterior fossa, pontocerebellar hypoplasia type 9, developmental delay, microcephaly pontocerebellar hypoplasia dyskinesia

## Abstract

Pontocerebellar hypoplasia type 9 (PCH9) is a rare, autosomal, recessive, neurodevelopmental disorder caused by a mutation in the AMPD2 gene. Despite its rarity, it presents distinctive clinical and neuroradiological features. Diagnosing it is challenging yet crucial for appropriate management. We describe a 21-month-old boy with clinical and neuroradiological manifestations of the diagnosis, including characteristic signs such as an eight-configured midbrain and hypoplasia of the brainstem and cerebellar structures. Genetic evaluation confirmed homozygous missense mutations in the AMPD2 gene. This case highlights the pathognomonic neuroradiological features of pontocerebellar hypoplasia type 9 that point toward diagnosis.

## Introduction

Pontocerebellar hypoplasias constitute a heterogeneous group of autosomal, recessive neurodevelopmental and neurodegenerative disorders with 19 associated genetic variants grouped into 13 disease subtypes [1]. Pontocerebellar hypoplasia type 9 (PCH9) is a rare subtype with unknown epidemiological data [2-4]. It is characterized by a unique combination of features caused by a genetic mutation in AMPD2 [5].

This represents the first reported case in Colombia with genetic confirmation of PCH9, exhibiting clinical and neuroradiological characteristics that should be considered in children with neurodevelopmental issues.

## Case presentation

**Figure 1:**
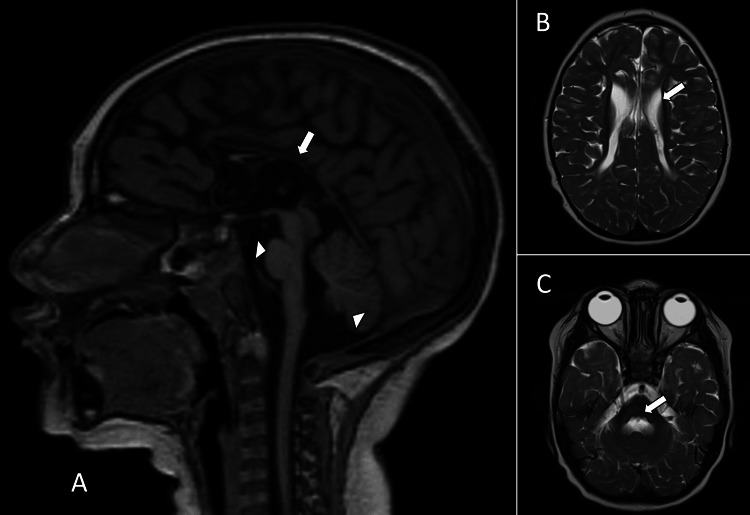
Brain magnetic resonance imaging without contrast administration (A) Sagittal T1-weighted volumetric image shows a decrease in the size of the cerebellum and less in the pons (white arrowheads) and hypoplasia of the corpus callosum (white arrow). (B) Axial T2-weighted image demonstrates significant loss of the periventricular white matter (arrow). (C) Axial T2-weighted image demonstrates pontine atrophy (white arrow).

A 21-month-old male patient, born as a result of a high-risk pregnancy, was delivered via emergent cesarean section due to prolonged labor. The delivery occurred at full term, with appropriate height and weight, and no reported neonatal adaptation issues or perinatal hospitalization. At one month of age, a simple cranial computed tomography scan was performed due to a pinpoint fontanelle and hypotonia, revealing hypoplasia of the corpus callosum (not shown). Subsequently, cerebral magnetic resonance imaging was performed, revealing colpocephaly indicative of white matter loss, resembling a periventricular leukomalacia, and resulting in a significant reduction in the size of the corpus callosum. The imaging also revealed hypoplasia of the brainstem and cerebellar structures (Figure 1).

**Figure 2:**
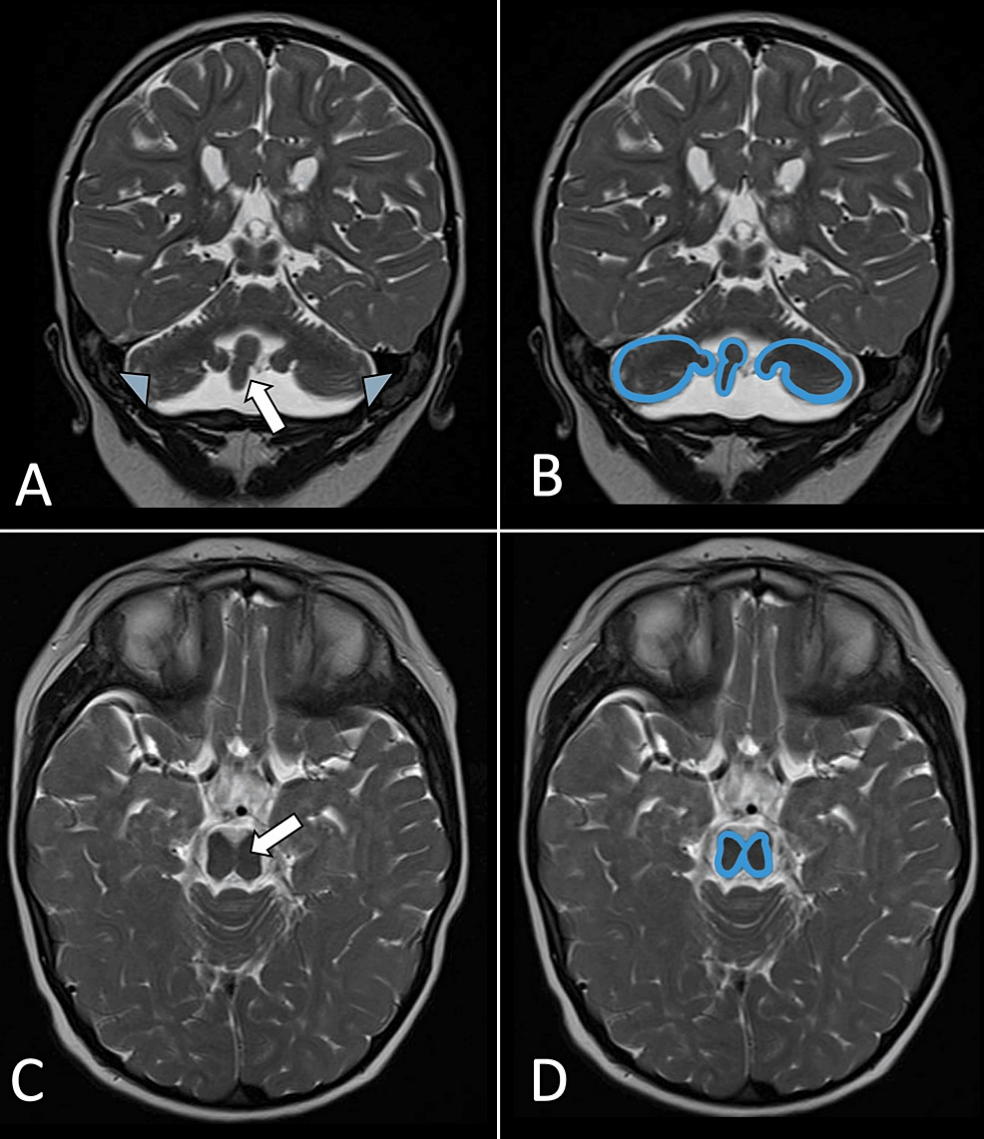
Brain magnetic resonance imaging without contrast administration (A) Coronal T2-weighted acquisition with the dragonfly appearance of the cerebellum; its wings are composed of atrophic cerebellar hemispheres (blue arrowheads) and a central vermis resembling a large body (white arrow). (B) Schematic example of the cerebellum shaped like a dragonfly or butterfly (C) Axial T2-weighted acquisition, the anatomical image of the midbrain in the shape of an 8 (arrow). (D) Schematic drawing of this finding. Illustrations by the authors

The hypoplasia of the cerebellar hemispheres was reminiscent of the shape of a dragonfly or butterfly in the coronal acquisition, whereas the brainstem atrophy exhibited a configuration resembling a “ mesencephalon in 8” in the axial acquisition (Figure 2).

Based on these findings, additional extensive studies and genetic evaluations were conducted. The patient underwent brainstem and visually evoked potential testing with flash, an echocardiogram, and an electroencephalogram, all of which yielded normal results. Exome sequencing with copy number variations revealed missense variants of uncertain significance in homozygosis in the AMPD2 gene. All findings were consistent with pontocerebellar hypoplasia type 9 (OMIM102771). Unfortunately, the patient was unable to follow up after diagnosis.

## Discussion

Pontocerebellar hypoplasias are a group of heterogeneous, autosomal recessive neurodevelopmental and neurodegenerative disorders with an estimated incidence of less than five cases per million [5]. This syndrome encompasses 19 genetic variants, which fall into 13 disease subtypes [1]. Due to its rarity, precise epidemiological frequencies are unknown; however, the incidence of the most common subtype, pontocerebellar hypoplasia type 2, is less than one in 200,000 births [6]. For the rarer variants, specific epidemiological data are lacking. To our knowledge, PCH9 has yet to be reported in Colombia [2,4-6].

PCH9 stems from a primarily missense mutation in the AMPD2 gene on chromosome 1p13. This gene encodes the adenosine monophosphate deaminase-2 (AMD2) enzyme, crucial for guanine nucleotide synthesis and protein translation, which play critical roles in neurogenesis. Neurons harboring mutations in AMPD2 experience guanine nucleotide depletion, likely resulting in adenosine accumulation with toxic effects. This accumulation initially restricts neuron growth and eventually leads to cell death. The regional differential expression of AMPD2, particularly in the cerebellum and pons, explains the correlations with areas of hypoplasia [2,3,5,7,8].

Patients with PCH9 exhibit a range of characteristics, including visual impairment, swallowing difficulties, truncal hypotonia, neonatal clonus, hyperreflexia, spasticity, progressive and severe microcephaly (up to -9 standard deviation), profound neurodevelopmental delay, axonal neuropathy, delayed myelination, and early-onset seizures. Certain dysmorphic features have been observed such as bitemporal narrowing, midface hypoplasia, a short upper lip, and macroglossia. Despite the name and the presence of cerebellar hypoplasia on imaging, cerebellar symptoms are rarely reported. In older patients, extrapyramidal involvement and axonal neuropathy may occur [2,3,5,7].

Neuroimaging reveals four pathognomonic characteristics that aid in syndromic diagnosis, particularly in cases with nonspecific clinical presentations. These features include progressive white matter alteration, resembling periventricular leukomalacia and leading to a significant reduction in the size of the corpus callosum, pontine hypoplasia, or atrophy; a cerebellum shaped like a dragonfly or butterfly in coronal acquisition due to marked atrophy of the cerebellar hemispheres with preserved vermis size; and a mesencephalon-in-8 configuration in axial acquisition. The mesencephalon-in-8 configuration is considered a pathognomonic sign, and, with the other characteristic findings, can lead to the diagnosis; however, it is not present in all cases [2,5]. When the pons hypoplasia is mild (as in our case) or in question, morphometric evaluation can help establish it [9,10]. Generalized atrophy of the brain cortex can also be associated with the condition [2-4,6,7,11-13]. The differential diagnoses are other pontocerebellar hypoplasias, metabolic, or other genetic diseases, where the neuroradiologist is key to directing the most probable diagnosis [2,6].

Despite recent advances and the promotion of purine nucleotide precursor administration, the management of PCH9 remains symptomatic and supportive. This approach focuses on addressing epilepsy, ensuring adequate nutritional intake, providing speech and physical therapy, and offering palliative care services. Due to the progressive and life-limiting nature of PCH9, the prognosis is poor, with most patients dying during childhood or early adolescence. Therefore, an accurate diagnosis is essential to provide directed care to the patient and offer counseling regarding the prognosis and genetic counseling to the families [8,14].

Our case report is limited because the patient received non-single institution management, which led to non-long-term follow-up in further understanding disease progression and management challenges. On the other side, this case report presents the first genetically confirmed patient with PCH9 in Colombia, contributing to the medical literature on the occurrence of this rare subtype in a previously unreported region and highlighting the associated neuroimaging structural abnormalities.

## Conclusions

The neuroradiological feature of mesencephalon-in-8 configuration is the key finding for PCH9, a rare and poorly understood disease in children with neurodevelopmental issues. Other imaging and clinical characteristics can help diagnose PCH9, which is confirmed by genetic identification of the mutated AMPD2 gene. Although management primarily focuses on symptomatic and supportive measures, accurate diagnosis is crucial for providing integral care with genetic guidance for families. Further research is needed to elucidate the long-term clinical and management complexities associated with PCH9.
